# Targeting the PI3K/AKT pathway: a potential new weapon in the global fight against SARS-CoV-2?

**DOI:** 10.7150/ijbs.63969

**Published:** 2021-07-05

**Authors:** Salvatore Santamaria

**Affiliations:** Department of Immunology and Inflammation, Imperial College London, Du Cane Road W12 0NN, London, UK.

**Keywords:** SARS-CoV-2, COVID-19, PI3K/AKT pathway

## Abstract

Commentary on 'Capivasertib restricts SARS-CoV-2 cellular entry: a potential clinical application for COVID-19' by Sun et al.

Severe acute respiratory syndrome coronavirus 2 (SARS-CoV-2) is a betacoronavirus responsible for coronavirus disease 2019 (COVID-19). Since its initial identification in 2020 1, the SARS-CoV-2 outbreak has developed into a pandemic that at the time of writing has costed the lives of at least 3.8 million people worldwide according to the World Health Organization (https://covid19.who.int/). Acute respiratory distress syndrome (ARDS) and multiple-organ failure are among the major causes of death 2. ARDS involves the accumulation of fluid in the lung and is characterised by elevated systemic levels of multiple cytokines ('cytokine storm') 3. Specific cytokines such as interleukin (IL)-1α, IL-1β, IL-6, IL-10, IL-18, tumour necrosis factor (TNF)-α, monocyte chemoattractant protein-1 (MCP-1) and interferon (IFN)-γ-induced protein (IP-10) have indeed been positively associated with the severity of SARS-CoV-2 infection 4.

In this issue of International Journal of Biological Sciences, Sun et al. describe an innovative approach to contrast SARS-CoV-2 infection which targets the downstream signalling cascades elicited by these cytokines 5. Following bioinformatic analysis, they first identified the phosphatidylinositol 3-kinase (PI3K)/protein kinase B (Akt) signalling pathway (PI3K/AKT) as the top-ranked kinase among those potentially associated with SARS-CoV-2 disease. To explore the potential targeting of this pathway as a SARS-CoV-2 therapy, they tested capivasertib (AZD5363), a pan-AKT kinase inhibitor, for its ability to inhibit infection of Vero cells by a SARS-CoV-2 spike protein-pseudotyped virus. Remarkably, capivasertib inhibited viral entry by 50% at ~2 μM, a concentration which authors have shown not to be cytotoxic. Capivasertib was not able to inhibit infection of the pseudovirion when the spike protein was deleted nor when this was overexpressed by Vero Cells rather than being present on the pseudovirion, thus suggesting that capivasertib was actually inhibiting viral entry.

Capivasertib can interfere with SARS-Cov-2 endocytic pathway by inhibiting activation of FYVE finger-containing phosphoinositide kinase (PIKFYVE) downstream of the PI3K/AKT signalling pathway, an activity that interferes with endocytic trafficking and, ultimately, with viral entry (**Figure 1**). Kinase inhibitors targeting the PI3K/AKT pathway have been previously shown to inhibit cell infection by Middle East respiratory syndrome coronavirus (MERS-CoV) 6. This shows that the PI3K/AKT pathway may be more generally involved in facilitating the infection of betacoronaviruses such as SARS-CoV-2 and MERS-CoV.

Perhaps not surprisingly, the downstream signalling cascades elicited by cytokines systemically increased in severe COVID-19 have been found to crosstalk with pathways involved in cancer 7 and in fact the present authors have shown that increased AKT1 mRNA levels correlated with poor prognosis in several types of cancer. The PI3K pathway is one of the most commonly activated signalling pathways in estrogen receptor-positive breast cancer 8. Capivasertib has recently been shown to increase progression-free survival in patients with aromatase inhibitor-resistant advanced breast cancer in a randomised, double-blind, placebo-controlled, phase 2 trial currently in follow-up status (clinical trial ID: NCT01992952) 9.

The data presented in the article by Sun. et al. may help to accelerate the repurposing of kinase inhibitors such as capivasertib for SARS-CoV-2 therapy. In view of this, it will be extremely informative to investigate the susceptibility to SARS-CoV-2 infection by non-vaccinated cancer patients subjected to treatment with pan AKT inhibitors. However, many questions still need an answer at the preclinical level. If capivasertib and, more generally, PI3K/AKT kinase inhibitors may prevent SARS-CoV-2 infection, at which stage they should be administered? Will PI3K/AKT kinase inhibitors be effective at low or high viral load or when a cytokine storm is already occurring? If PI3K/AKT kinase inhibitors are effective only when virus titre is low, then it may not be feasible to administer them to a large asymptomatic SARS-CoV-2-infected population, since in the non-randomised NCI-MATCH study (clinical trial ID: NCT00700882), administration of capivasertib was discontinued in 31% of patients due to toxicity issues 10. Whatever story the future preclinical studies will tell, the administration of PI3K/AKT kinase inhibitors for COVID-19 management should be finely tuned to balance therapeutic benefits.

## Figures and Tables

**Figure 1:**
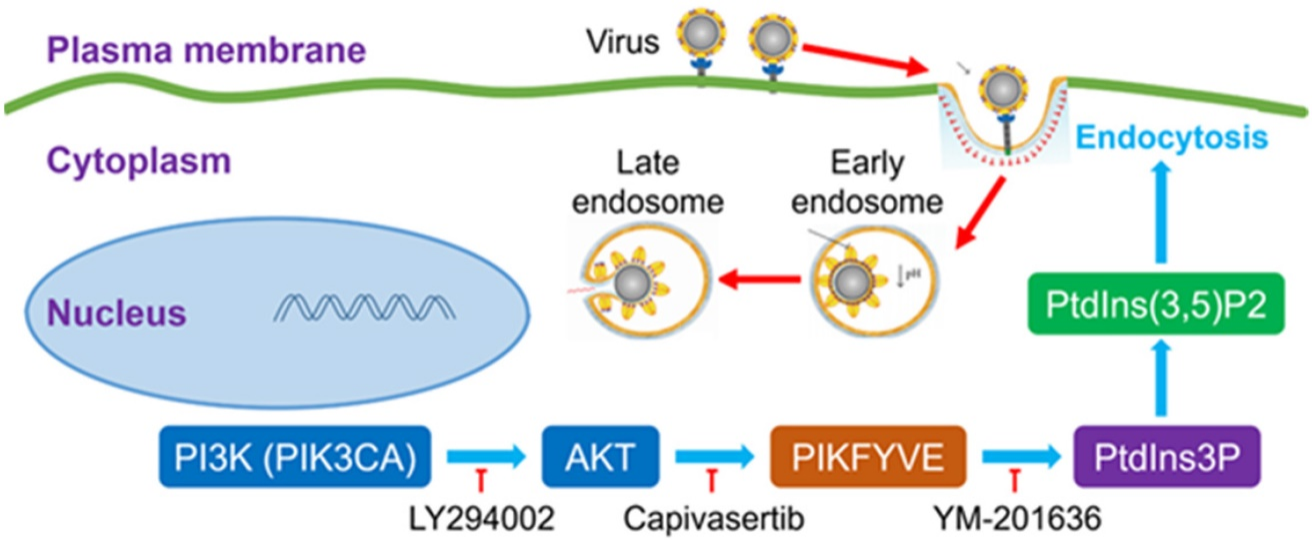
** Potential mechanism of action of PI3K/AKT kinase inhibitors on SARS-CoV-2 viral entry.** Capivasertib inhibits activation of FYVE finger-containing phosphoinositide kinase (PIKFYVE) by AKT kinases, thus interfering with endocytic trafficking and, potentially, SARS-CoV-2 viral entry. Other drugs potentially effective against SARS-CoV-2 infection include LY294002, a broad-spectrum inhibitor of PI3K upstream of PIKFYVE, and YM-201636, which inhibits phosphorylation of PtdIns3P to PtdIns(3,5)P2 by PIKFYVE (Courtesy of the authors, Sun et al. 5).
